# Nano-Dispersed Ziegler-Natta Catalysts for 1 μm-Sized Ultra-High Molecular Weight Polyethylene Particles

**DOI:** 10.3389/fchem.2018.00524

**Published:** 2018-10-30

**Authors:** Patchanee Chammingkwan, Yusuke Bando, Minoru Terano, Toshiaki Taniike

**Affiliations:** Japan Advanced Institute of Science and Technology, Graduate School of Advanced Science and Technology, Nomi, Japan

**Keywords:** nano-sized catalyst, ziegler-natta, nano-dispersed, magnesium oxide, polyethylene, ultra-high molecular weight, microfine

## Abstract

A catalytic approach to synthesize microfine ultra-high molecular weight polyethylene (UHMWPE) particles was proposed based on the exploitation of nano-sized catalysts. By utilizing MgO nanoparticles as a core material, a Ziegler-Natta-type MgO/MgCl_2_/TiCl_4_ core-shell catalyst with the particle size in a nano-range scale was prepared in a simple preparation step. The organic modification of MgO surfaces prior to catalyzation prevented agglomeration and facilitated the full dispersion of catalyst particles at a primary particle level for the first time. The nano-dispersed catalysts successfully afforded a direct access to UHMWPE having the particle size in the range of 1–2 μm at a reasonable activity. Extremely fine polymer particles yielded several advantages, especially at a significantly lower fusion temperature in compression molding.

## Introduction

Polyethylene (PE) having a molecular weight over 1–1.5 × 10^6^ g mol^−1^, termed as ultra-high molecular weight polyethylene (UHMWPE), equips a range of advantages over commodity high-density PE (HDPE), such as high abrasion resistance, excellent impact toughness, good corrosion and chemical resistance, resistance to cyclic fatigue and radiation, and self-lubricating ability (Kurtz, 2014). As a result, it has been highly demanded in numerous applications, especially under harsh environment. While a number of catalysts have been developed and disclosed [non-supported or supported metallocene, fluorinated-oxide supported chromium, etc. (Alt and Köppl, 2000; Matsui and Fujita, 2001; Furuyama et al., 2003; Weiser and Mülhaupt, 2006; Jones and Armoush, 2009; Jones et al., 2010; Mihan et al., 2011)], the industrial production of UHMWPE is dominantly owed by heterogeneous Ziegler-Natta catalysts, MgCl_2_/TiCl_4_. Prohibitively high melt viscosity of UHMWPE necessitates direct processing of as-synthesized reactor powder, in which the initial morphology of UHMWPE powder is retained in the final product to be a cause of abrasive wear and ultimate failure (Farrar and Brain, 1997; Smith et al., 2011; Bracco et al., 2017).

Various methods have been proposed to improve the processability of UHMWPE. This includes the addition of low molecular weight PE (Takahashi et al., 1995) or liquid paraffin (Liu et al., 2015) as a processing aid. However, some or considerable losses of the beneficial properties of UHMWPE are accompanied due to the dilution with the low molecular weight component and shear degradation in the extruder (Takahashi et al., 1995). Catalytic approaches have been also adopted to produce UHMWPE featured with enhanced flow characteristics and drawability. These methods target at minimizing the density of polymer chain entanglement as it restricts the mobility of polymer. Ethylene is polymerized under a condition that a single polymer chain crystallizes without overlapping with the others. This requires diluted active sites and low temperature for the crystallization, especially polymerization using a diluted molecular catalyst for a spatial distance between growing alkyl chains sufficiently far to allow chain folding into individual crystals as soon as they come out (Pandey et al., 2011; Rastogi et al., 2011). Recently, a compartmentalization concept has been applied to a heterogeneous catalyst to provide the spatial distance between the growing chains. For example, Li et al. employed polyhedral oligomeric silsesquioxane (POSS) as a spacer in a fluorinated bis(phenoxyimine)Ti dichloride/SiO_2_ catalyst system (Li et al., 2016). POSS was bonded to a methylaluminoxane-activated SiO_2_ surface, and this became a horizontal spacer to compart the neighboring active sites and disallow an overlap of polymer chains. Another work from the same group showed that POSS can capture MgCl_2_ through its hydroxyl groups to form nano-sized aggregates that served as a spacer in a TiCl_4_/MgCl_2_/SiO_2_ catalyst system for the synthesis of weakly entangled UHMWPE with enhanced flow properties (Li et al., 2018).

The simplest yet effective route to improve processability of UHMWPE is to control/reduce the size of polymer particles as building blocks (Baumgaertner, 1974; Han et al., 1981; Barnetson and Hornsby, 1995). Though several processes, such as emulsion polymerization and thermally induced phase separation, are commonly applied to access polymer fine particles (Ogawa et al., 2007; Rudin and Choi, 2013), these processes are hardly applicable to UHMWPE owing to the catalyst deactivation in the presence of a polar solvent as well as the limitation of polymer dissolution in a common organic solvent. Consequently, a direct approach to obtain fine particles in catalyzed olefin polymerization is most preferred. Known as replication phenomena (Taniike et al., 2011; Soares and McKenna, 2012), the particle size and its distribution of UHMWPE reactor powder are determined by those of the catalysts employed, in which the final polymer particle size is roughly proportional to *Y*^1/3^ *D*_Cat_, where *Y* and *D*_Cat_ represent the polymer yield per gram-catalyst and the size of catalyst particles, respectively. Considering not only the essential requirement for a low impurity level in the obtained polymer, but also the factor of 1/3 for *Y*, the range of the polymer particle size controllable by the polymer yield is quite limited. Hence, minimizing the catalyst particle size without scarifying the activity and morphological integrity during polymerization is essential. In general, Ziegler-Natta catalysts possessing particle sizes around 5–20 μm are used to produce commodity grades of UHMWPE having particle sizes of 100–300 μm. Whilst, microfine particles (e.g., below 80 μm) can be accessed by catalysts with the sizes of a few-to-several micrometers (Suga et al., 1990). A number of preparation protocols have been developed to access a Ziegler-Natta catalyst possessing a small particle size. However, most of the processes necessitate complicated chemical formulas as well as technically demanding processes. For example, a multistep process is typically required for the preparation of Mg solution precursors, and subsequent precipitation/titanation to form morphologically controlled solid particles (Cuffiani and Zucchini, 1996; Bilda and Boehm, 2000; Kidd et al., 2010). In some cases, a high speed shearing either during the formation of a solid support or after forming a solid catalyst (Suga et al., 1990; Nakayama et al., 2009) is further required to bring the particle size down to a few-to-several micrometers. To escape from an elaborate preparation process, the development of catalyst preparation protocol to access small catalyst particles in a simple manner is still highly desired. Especially, a Ziegler-Natta catalyst with the particle size in a nano-range scale is expected to facilitate the greatest advantage for *Y*^1/3^ *D*_Cat_.

A simple protocol for the preparation of a Ziegler-Natta nanocatalyst was developed by our group using MgO nanoparticles as a core material (Taniike et al., 2012a,b; Chammingkwan et al., 2014). In this catalyst system, catalyzation proceeds on the surfaces of MgO cores: The chlorination using TiCl_4_ converts MgO outermost surfaces into a thin MgCl_2_ overlayer, and simultaneously immobilizes TiCl_4_ on MgCl_2_ via chlorine bridges to form a core-shell MgO/MgCl_2_/TiCl_4_ catalyst in one step. The catalyst possesses a similar active site nature to a typical Ziegler-Natta catalyst, thus being active for olefin polymerization. Recently, we have successfully applied the core-shell MgO/MgCl_2_/TiCl_4_ catalyst for the synthesis of UHMWPE with a reasonable activity (Bando et al., 2018). However, it was found that the size of polymer particles did not reflect the nano-sized nature of the catalyst particles. The key issue was found at the dispersion of nanoparticles: MgO was poorly dispersed in a non-polar solvent due to its hydrophilic nature, hence causing the agglomeration of catalyst particles. While an organic modification of MgO surfaces helped to alleviate the dispersion problem, the removal of the organic modifier from MgO surfaces by TiCl_4_ prevented the exploitation of nano-sized features for polymer particle control. In this work, we attempted to screen a variety of organic modifiers to access truly nano-dispersed Ziegler-Natta catalyst particles. By a careful selection of the organic modifier, a catalyst with the dispersion at a nano-level was attained for the first time, which enabled the direct synthesis of extremely fine UHMWPE particles featured with several advantages in compression molding.

## Material and methods

### Materials

MgO nanoparticles with the mean particle size of 50 nm (Wako Pure Chemical Industries Ltd.), 100 nm (Alfa-Aesar), and 200 nm (Wako Pure Chemical Industries Ltd.) were used after dehydration at 160°C under vacuum for 2 h. Titanium tetrachloride (TiCl_4_) and kerosene of research grade were used as received. Sorbitan monooleate (C_24_H_44_O_6_, donated by Kao Co.), sorbitan sesquioleate (C_33_H_60_O_6.5_, donated by Kao Co.), methyl oleate (C_19_H_36_O_2_, Tokyo Chemical Industry Co., Ltd.), and polyoxyethylene alkylamine (RN(C_2_H_4_O)_x_(C_2_H_4_O)_y_, donated by NOF Co.) were used for the organic modification of MgO surfaces (the chemical structures are given in Figure S1). *n*-Heptane was used after dehydration by passing through a column of molecular sieve 4A, followed by N_2_ bubbling for 2 h. Ethylene of polymerization grade was purchased from Hokurikuekikasangyou Co., Ltd. and used as received. Triethylaluminium (TEA, donated by Tosoh Finechem Co.) was used after dilution in heptane. A precipitation-based Ziegler-Natta catalyst (denoted as R-Cat, *D*_50_ = 7.95 μm, donated by IRPC Public Co., Ltd.) was used as a reference catalyst in ethylene polymerization.

### Surface modification of MgO and catalyst preparation

To a suspension of 10 g of dehydrated MgO powder in 25 mL of kerosene, 8 mL of an organic modifier was added. The mixture was heated at 160°C for 1 h under stirring at 250 rpm. The product was repetitively washed with heptane to yield organically modified MgO. The samples were named as X-MgOY, where X corresponded to the type of the organic modifier (sorbitan monooleate = SM, sorbitan sesquioleate = SS, methyl oleate = MO, and polyoxyethylene alkylamine = PA), and Y corresponded to the particle size of employed MgO.

2.0 g of PA-MgO was treated with 30 mL of TiCl_4_ in 100 mL of heptane at the reflux temperature for 1 h. Thus obtained catalyst was repetitively washed with heptane and kept as a slurry in heptane. The samples were named as PA-Cat50, PA-Cat100 and PA-Cat200. Reference catalyst samples were also prepared from pristine MgO powder having the particle size of 50 and 200 nm according to the same procedure. The samples were named as Cat50 and Cat200, respectively.

### Polymerization

Ethylene polymerization was performed in a 1 L stainless steel reactor equipped with a mechanical stirrer rotating at 500 rpm. After sufficient N_2_ replacement, 500 mL of heptane as a polymerization medium and 1.0 mmol of TEA as an activator were introduced. The solution was then saturated with 0.8 MPa of ethylene at 70°C for 30 min. Ten milligram of a catalyst was injected to start polymerization. The polymerization was carried out at 70°C under 0.8 MPa of ethylene pressure for 2 h. Thus obtained polymer was filtered and dried in vacuum at 60°C for 6 h.

### Characterization

Images of MgO nanoparticles were recorded on a transmission electron microscope (TEM, Hitachi H-7100) operated at an accelerate voltage of 100 kV. MgO powder was dispersed in ethanol, drop-casted on a copper grid, and naturally dried. The particle size distribution profiles of MgO and catalyst samples were acquired from light scattering (Horiba Partica LA-950V2). The measurements were done in a suspension form using heptane as a medium unless stated. The particle size was expressed as *D*_10_, *D*_50_, and *D*_90_, which corresponded to the particle size at 10, 50, and 90% of the cumulative volume distribution. A relative span factor (RSF) was calculated based on Equation (1):

(1) $$\begin{array}{l} \text{RSF }=\;\frac{D_{90}\;-\;D_{10}}{D_{50}} \end{array}$$

The presence of the organic modifier on MgO surfaces was observed by attenuated total reflectance infrared spectroscopy (ATR-IR, Perkin Elmer Spectrum 100 FT-IR) in the range of 4,000–500 cm^−1^. The Ti content of a catalyst was determined by ultraviolet-visible spectrometry (UV-vis, Jasco V670). Fifty milligram of the catalyst was dissolved in an aqueous solution of hydrochloric acid and sulfuric acid. Thereafter, 200 μL of hydrogen peroxide (35% aqueous solution) was added. The Ti content was determined based on the intensity of the absorption band at 410 nm (Taniike et al., 2014).

The particle size of polymer reactor powder was acquired by light scattering using ethanol as a medium. The observed particle size was compared with the theoretical size estimated according to Equation (2):

(2) $$\begin{array}{l} D_{\text{PE}}\;=\;{\left(\frac{d_{\text{Cat}}}{d_{\text{PE}}}Y\right)}^{\frac{1}{3}}D_{\text{Cat}} \end{array}$$

where *d*_Cat_ and *d*_PE_ are the densities of the catalyst and polymer, respectively. *Y* is the polymerization yield in g-PE g-Cat^−1^. *D*_PE_ and *D*_Cat_ are the particle sizes of the polymer and catalyst, respectively. This equation assumes that one polymer particle is formed per one catalyst particle without disintegration of the catalyst particle during polymerization. The particle size of polymer in a dry form was also measured based on an image analysis of vacuum-dispersed powder using a VD-3200 nano-particle size analyzer (JASCO). A vacuum-type dispersion unit allowed the dispersion of polymer particles on a glass plate in a dry state, and the particle characteristics were acquired by an automatic image analysis using Pro image analysis software. The viscosity-average molecular weight (*M*_*v*_) of polymer was determined based on ASTM D4020. This method is widely used to acquire the molecular weight of UHMWPE as it is nearly impossible to apply gel permeation chromatography due to restriction of solution flow and shear-induced chain scission in the column. The viscosity of a diluted polymer solution in decahydronaphthalene was measured at 135°C using an Ubbelohde-type viscometer, and *M*_*v*_ was derived from the intrinsic viscosity of polymer ([η]) according to Equation (3):

(3) $$\begin{array}{l} M_{v}\;=\;5.37\;\times {\;10}^{4}{\left[\eta \right]}^{1.37} \end{array}$$

The melting temperature (*T*_m_) and crystallization temperature (*T*_c_) of polymer were acquired using a differential scanning calorimeter (DSC, Mettler Toledo DSC 822). As-obtained reactor powder was heated to 180°C at the heating rate of 10°C/min under N_2_ flow. *T*_m_ of polymer for the nascent form was obtained from the melting endotherm in the first heat cycle. After holding at 180°C for 10 min, the sample was cooled down to 50°C at the cooling rate of 10°C/min to acquire non-isothermal *T*_c_. Thereafter, the second heat cycle was applied at the heating rate of 10°C/min to obtain *T*_m_ for the melt-crystallized form.

### Compression molding

Reactor powder was molded into a 5 cm × 5 cm specimen with the thickness of 500 μm by compression molding using a flash picture-frame mold. A specified amount of reactor powder was filled into an aluminum chase sandwiched between two thin ferrotype plates, and pressed with a contact pressure at room temperature for 5 min. Thereafter, the temperature was raised to a molding temperature and kept for 6 min before applying full pressure of 20 MPa for additional 5 min. The specimen was then cooled to room temperature. Different molding temperatures in the range of 120–140°C were applied to observe the initiation of fusion. As-obtained reactor powder was also used to form a scratch resistant coat. On a 5 cm × 5 cm HDPE plaque, a specified amount of reactor powder was placed, followed by compression molding using the above mentioned procedure at the molding temperature of 140°C.

## Result and discussion

**Figure 1:**
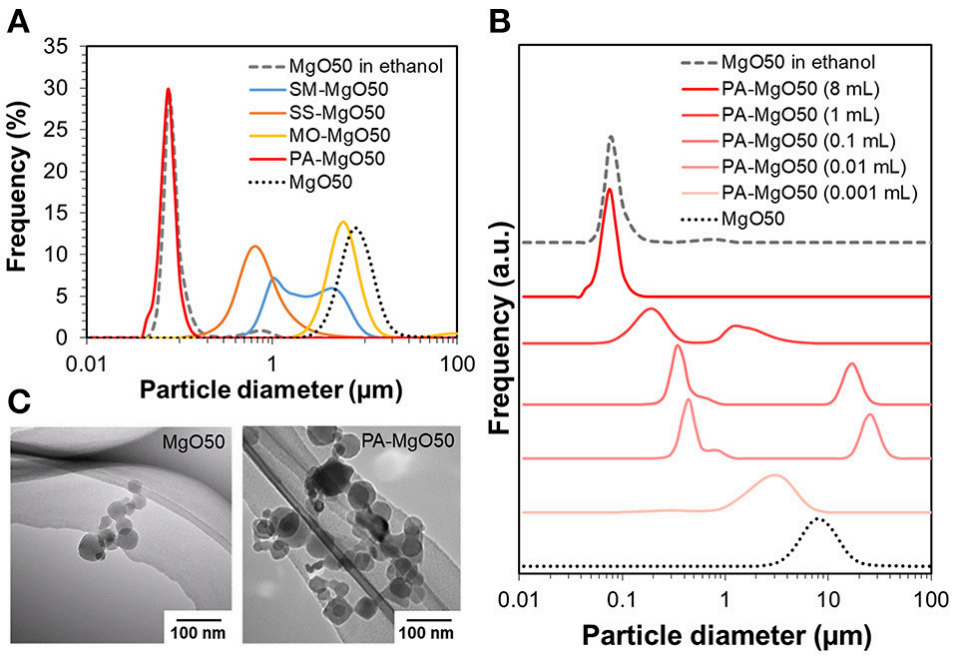
Particle size distribution profiles of MgO50 before and after treating with different types of organic modifiers **(A)**, and different amounts of polyoxyethylene alkylamine **(B)**. The analysis was conducted as a suspension in heptane unless stated. TEM images of pristine MgO50 and PA-MgO50 **(C)**.

A nano-sized Ziegler-Natta catalyst was developed based on the utilization of MgO nanoparticles as a core material. Since a non-polar solvent is required in catalyzation, full dispersion of MgO in the medium is essential to prevent the agglomeration of catalyst particles. As shown in Figure 1A, MgO50 was highly dispersible in ethanol with a sharp particle size distribution profile. The mode size was ca. 80 nm, and was small enough to be regarded as the dispersion of primary particles. Contrary, the same sample was poorly dispersed in heptane as demonstrated in the mode size of around 10 μm. Organic modifiers of various types in the group of non-ionic surfactants were applied for the surface modification (Figure 1A). It should be noted that these organic modifiers have a similar length of aliphatic chains, while different functional groups are present in the head group (see Figure S1). In all cases, the adsorption is expected to occur through hydrogen bonding between the functional group and hydroxyl groups available on MgO surfaces. Light scattering results showed that the treatment with the organic modifiers caused the shift of the particle size toward the primary particle size, while only polyoxyethylene alkylamine afforded fully dispersible MgO. These results indicated a different strength of the adsorption, in which multiplicity of anchoring groups is important to attain the strong adsorption. Figure 1B and Table 1 show the light scattering results of MgO50 that was treated with different amounts of polyoxyethylene alkylamine. The increase in the addition amount from 0.001 to 1 mL caused a change in the particle size distribution profile in a non-uniform way: Unimodal and bimodal distribution profiles appeared. However, the particle size always shifted toward the primary particle size, and this was true for the particle size in the first mode of the bimodal distribution. By further increasing the amount of polyoxyethylene alkylamine to 8 mL, MgO nanoparticles became fully dispersed at a primary particle level. These resulted implied that polyoxyethylene moiety helped to eliminate the attraction force among nanoparticles and/or to endow nanoparticles with surface hydrophobicity, in which a full surface coverage was essential for the homogenous dispersion of primary particles. TEM images in Figure 1C confirmed that a polygonal morphology of MgO nanoparticles was well-preserved after the surface modification.

**Table 1:** Particle characteristics of organically modified MgO.

**Sample**	**Particle size**^**a**^ **(**μ**m)**			**RSF^b^**
	***D*_10_**	***D*_50_**	***D*_90_**
MgO50	4.48	7.58	13.0	1.12
PA-MgO50 (0.001 mL)	1.08	2.50	4.54	1.38
PA-MgO50 (0.01 mL)	0.354	0.699	27.9	39.4
PA-MgO50 (0.1 mL)	0.283	0.492	19.2	39.5
PA-MgO50 (1 mL)	0.119	0.230	2.00	8.18
PA-MgO50 (8 mL)	0.054	0.070	0.088	0.490
MgO50 in ethanol	0.061	0.077	0.120	0.766

a Analyzed by light scattering as a suspension in heptane unless stated.

b *Calculated based on Equation (1)*.

**Figure 2:**
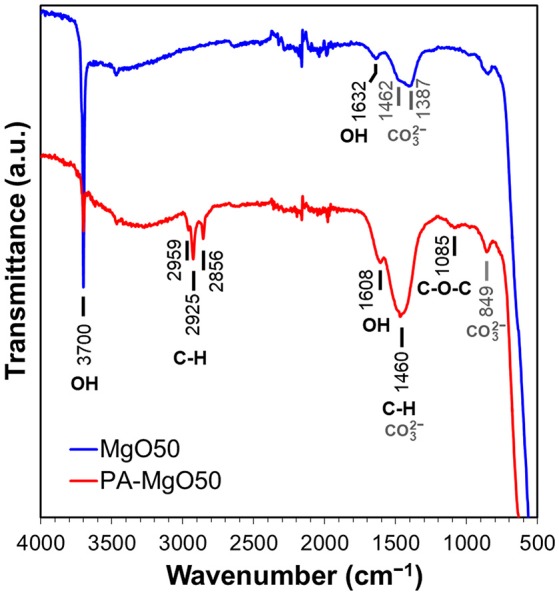
ATR-IR spectra of PA-MgO50, referenced to pristine MgO50.

ATR-IR spectra were acquired to confirm the presence of polyoxyethylene alkylamine on MgO surfaces (Figure 2). In the spectrum of MgO50, a sharp peak at 3,700 cm^−1^ indicates the presence of physisorbed water, which also accompanies the OH bending at 1,632 cm^−1^ (Todan et al., 2014; Li et al., 2017). The bands between 1,300–1,500 cm^−1^ are assigned to the O-C-O vibration from CO_2_ impurity adsorbed on MgO surfaces in different modes of the adsorption (Fukuda and Tanabe, 1973; Teramura et al., 2004). The band at 849 cm^−1^ is ascribed to the C = O vibration of the bidentate carbonate complex of CO_2_ (Yanagisawa et al., 1995; Teramura et al., 2004). In the spectrum of PA-MgO50, new bands belonging to the organic modifier were observed. The peaks at 2,959, 2,925, and 2,856 cm^−1^ are assigned to the asymmetric stretching of CH_3_, asymmetric stretching of CH_2_, and symmetric stretching of CH_2_ from the aliphatic chain, respectively (Colthup et al., 1975). A broad band around 1,085 cm^−1^ corresponds to the asymmetric C-O-C stretching of the repeating -O-CH_2_-CH_2_-O- units of polyoxyethylene (Tanaka and Igarashi, 2004), while an intense peak at 1,460 cm^−1^ comes from both of the CO_2_ adsorption (Li et al., 2017), and the CH_2_ deformation bending (Colthup et al., 1975).

**Figure 3:**
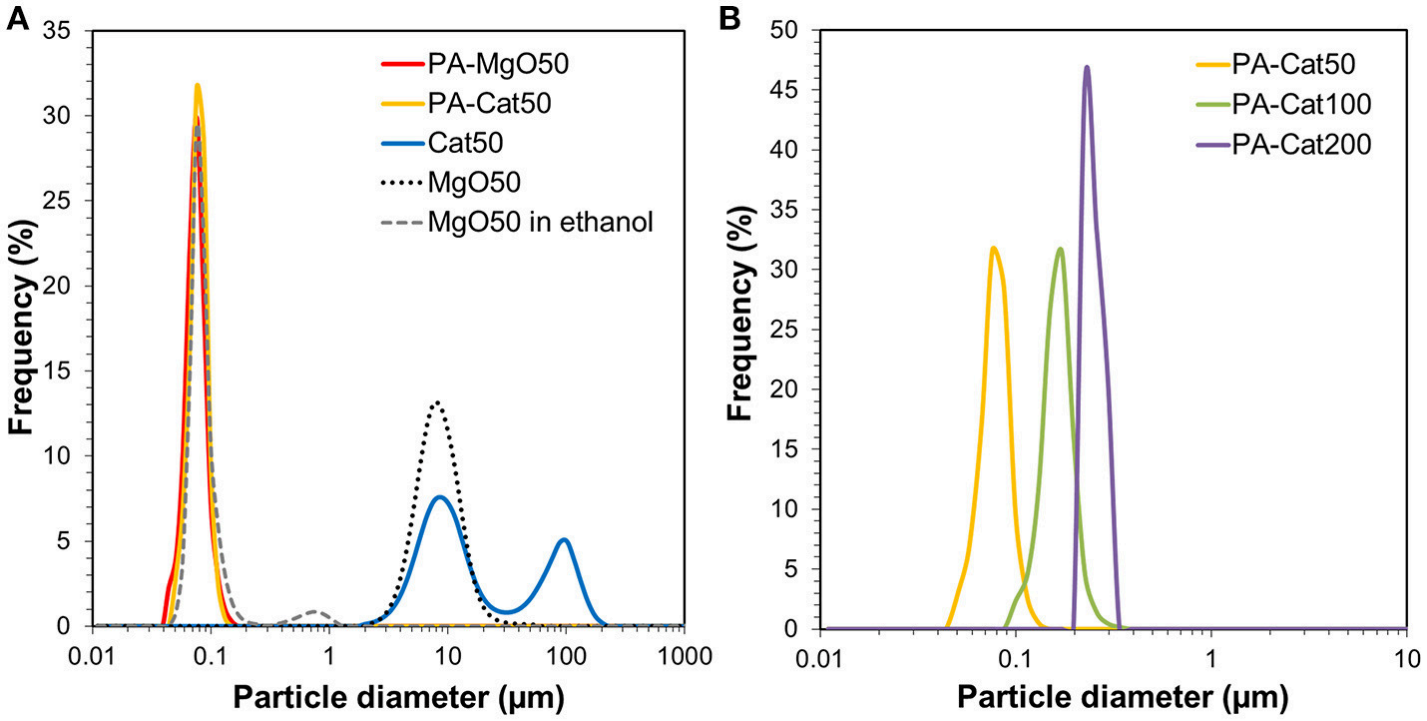
Particle size distribution profiles for MgO50 and before and after catalyzation **(A)**, and those for catalyst samples prepared from organically modified MgO having different particle sizes **(B)**.

Figure 3A illustrates the light scattering results of PA-MgO50 and MgO50 before and after catalyzation. The particle characteristics are also summarized in Table 2. In the case of pristine MgO, the catalyzation further promoted the agglomeration, where a second peak in the particle size distribution profile appeared at around 100 μm (Cat50). As mentioned earlier, such a severe agglomeration was originated from poor dispersion of MgO in a non-polar solvent. Suspended nanoparticles were electrostatically agglomerated. Once a thin layer of MgCl_2_ was formed, the agglomeration further progressed and became irreversible due to an enhanced attraction arising from an ionic character and/or the formation of a hard neck at the contact points. When the surface modification was applied (PA-Cat50), the agglomeration during catalyzation could be fully prevented due to the presence of adlayer. We also attempted to apply the same procedure to MgO nanoparticles having different particle sizes and the results showed that all of the catalysts became fully dispersible at the primary particle level (Figure 3B). Hence, the proposed method offered an access to nano-dispersed Ziegler-Natta catalysts, whose size could be easily controlled through the size of original MgO nanoparticles.

**Table 2:** Particle characteristics of catalysts.

**Sample**	**Particle size**^**a**^ **(**μ**m)**			**RSF^b^**
	***D*_10_**	***D*_50_**	***D*_90_**
PA-Cat50	0.058	0.073	0.090	0.438
PA-Cat100	0.118	0.153	0.191	0.477
PA-Cat200	0.203	0.230	0.279	0.330
Cat50^c^	5.02	11.3	98.3	8.25
Cat200^c^	3.32	5.95	10.0	1.12

a Analyzed by light scattering as a suspension in heptane.

b Calculated based on Equation (1).

c *Prepared from pristine MgO*.

The polymerization performance of the nano-dispersed catalysts was examined and compared with reference catalysts (Cat50, Cat200, and R-Cat). In Table 3, the Ti content and the polymerization activity increased with the decrease in particle size of MgO for both of the modified and non-modified catalyst systems. However, it could be recognized that the activities for the modified catalysts were at maximum halved from that of non-modified ones. Considering that the particle size distribution profile of the nano-dispersed catalysts was maintained at the primary particle level, it was most plausible that the organic modifier retained on the surfaces during chlorination. The presence of electron donating groups as well as steric restriction upon chlorination might restrict the activity. Nonetheless, it must be mentioned that the catalyst efficiency per Ti content of PA-Cat50 was higher than that of a typical precipitation-based Ziegler-Natta catalyst (R-Cat), while the Cl content in the resultant polymer was estimated to be an order of a magnitude lower due to the Cl existence only in the thin MgCl_2_/TiCl_4_ catalytic layer (below 2 nm) (Chammingkwan et al., 2014). Simplicity in the catalyst preparation featured with the reasonable activity as well as a reduced Cl residue in polymer powder made nano-dispersed MgO/MgCl_2_/TiCl_4_ catalysts promising for an industrial application.

**Table 3:** Polymerization results.

**Sample**	**Ti content^a^** **(wt%)**	**Activity^b^** **(g-PE g-Cat^−1^)**	**Polymer particle characteristics**		
			**$D_{50}^{\text{c}}$** **(μm)**	**RSF^d^**	**Theoretical size^e^** **(μm)**
PA-Cat50	0.76	3200	77.3	0.564	1.67
PA-Cat200	0.33	68	171	0.370	1.46
Cat50	0.47	6240	711	1.01	324
Cat200	0.17	420	633	0.870	69
R-Cat^f^	2.5	7900	147	0.686	212^g^

a Determined based on UV-vis spectroscopy.

b Polymerization conditions: Ethylene pressure = 0.8 MPa, heptane = 500 mL, TEA = 1.0 mmol, catalyst = 10 mg, T = 70°C, t = 2 h.

c Analyzed by light scattering as a suspension in ethanol.

d Calculated based on Equation (1).

e The theoretical polymer particle size was calculated based on Equation (2). The densities of polymer and the catalysts were set to 0.97 g cm^−3^ for UHMWPE and 3.65 g cm^−3^ for MgO, respectively. The catalyst particle size in Equation (2) was set to the D_50_ value acquired from light scattering (cf. Table 2).

f A precipitation-based Ziegler-Natta catalyst (D_50_ = 7.95 μm) was supplied from IRPC Public Co., Ltd.

g The density of R-Cat was set at 2.32 g cm^−3^ for MgCl_2_.

**Figure 4:**
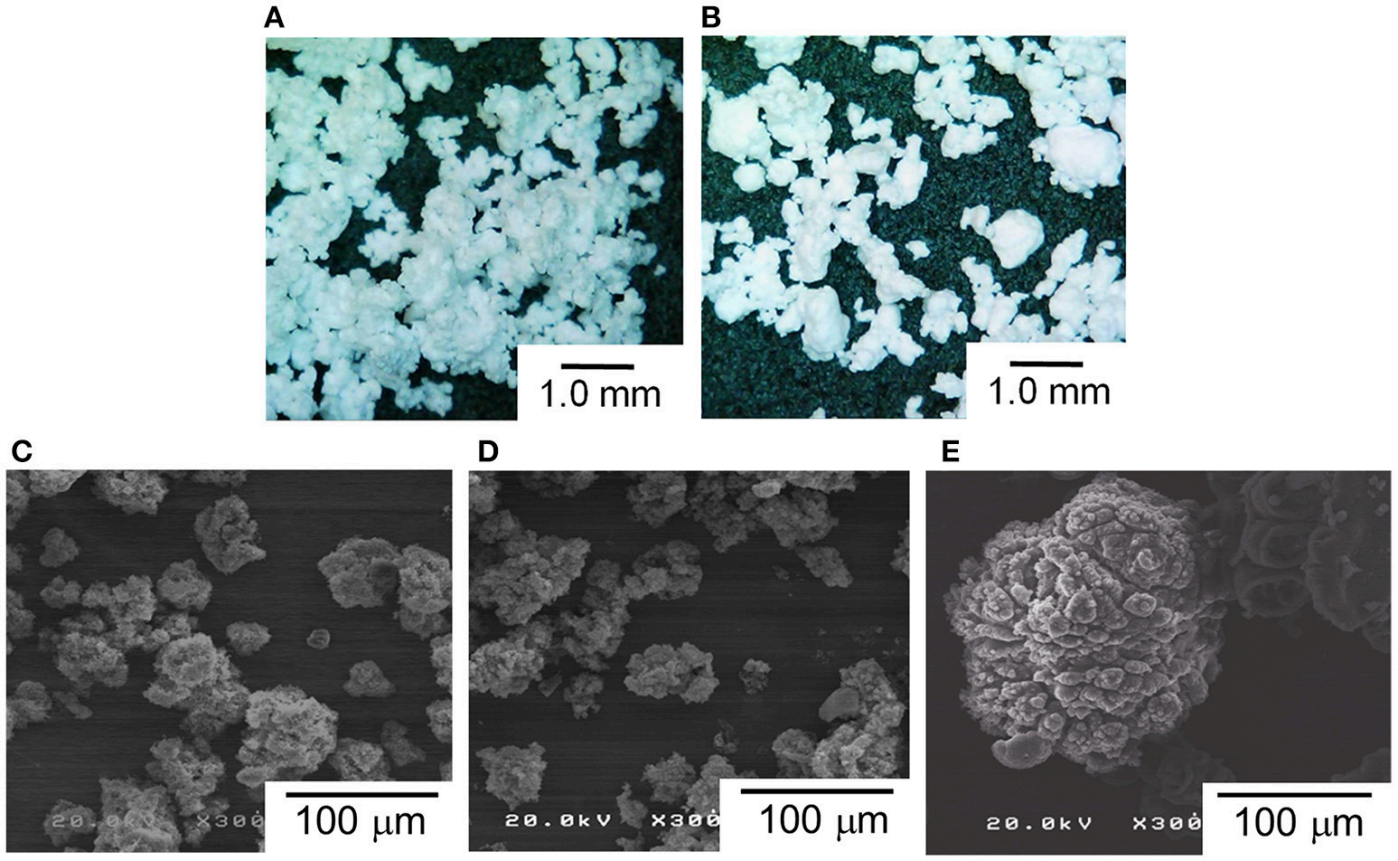
Morphology of polymer reactor powder: microscope images of PE50 **(A)**, and PE200 **(B)**. SEM images of PA-PE50 **(C)**, PA-PE200 **(D)**, and R-PE **(E)**.

**Figure 5:**
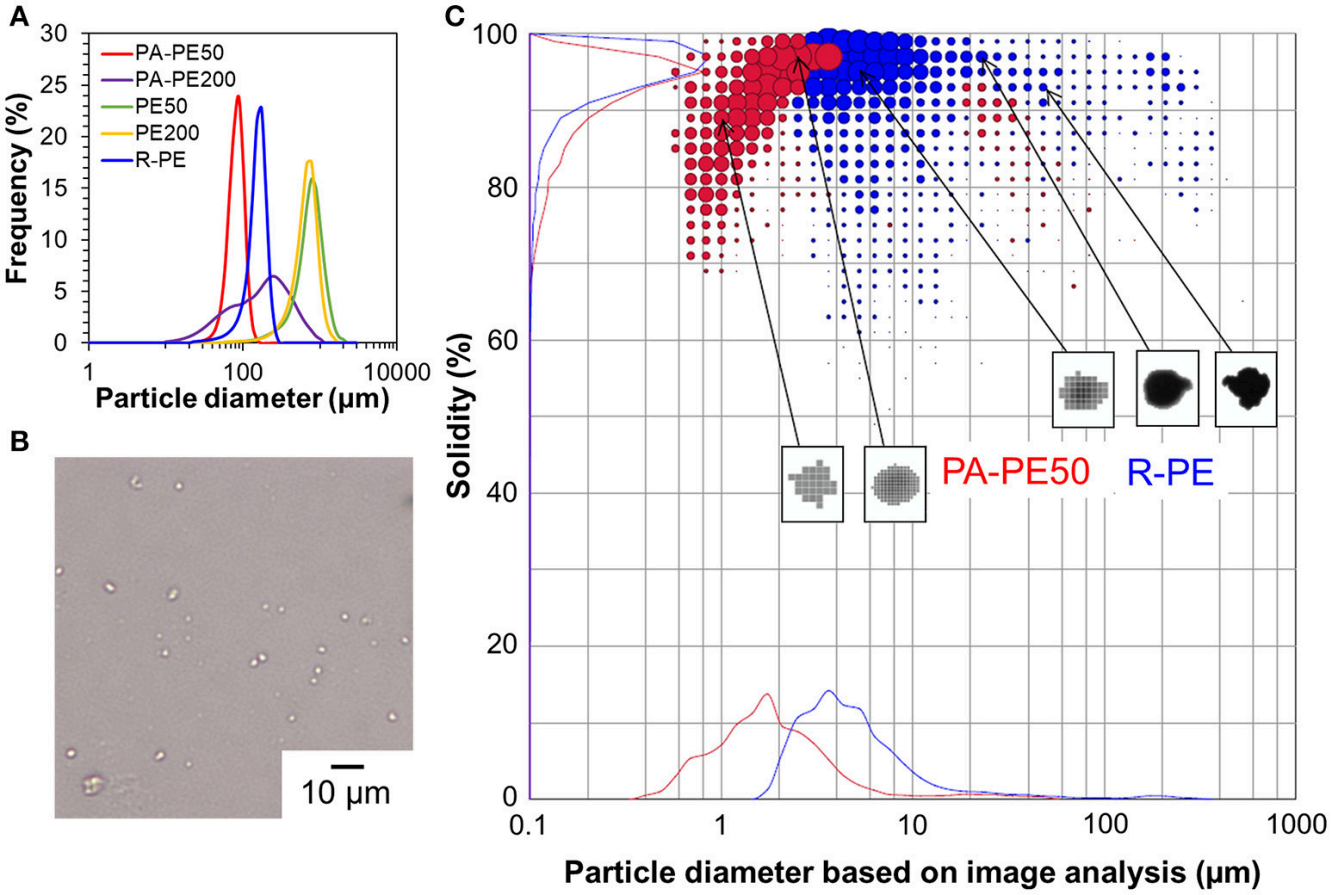
Polymer particle characteristics: particle size distribution profiles of polymer reactor powder in ethanol **(A)**, microscope image of PA-PE50 dispersed on a glass plate **(B)**, and particle characteristics based on an image analysis of vacuum-dispersed polymer particles **(C)**.

The morphology of polymer reactor powder was observed either by an optical microscope or SEM, depending on the particle characteristics. In the absence of the surface modification, as-obtained polymer powder apparently exhibited chunk-like and non-free-flow characteristics (PE50 and PE200). Optical microscope images of these samples show a coarse body of heavily agglomerated structures (Figures 4A,B). Contrary, free-flow polymer powder was obtained for the modified catalysts. SEM images (Figures 4C,D) show that the particle sizes for PA-PE50 and PA-PE200 were much smaller than those obtained from the non-modified system. Microscopically, each particle was composed of a random agglomeration of many small particles. On the other hand, R-Cat gave polymer with a popcorn-like morphology, which is typical for a multigrain catalyst (Figure 4E; Kissin et al., 2017). The particle sizes were acquired by light scattering in ethanol (Figure 5A) and the results are compared with the theoretical sizes. From Table 3, R-Cat gave polymer with a smaller particle size as compared to the theoretical size. Bearing in mind that that Equation (2) assumes a dense sphere for both of the catalyst and polymer particles, the deviation between the observed and theoretical sizes for R-Cat was originated from its porous structure rather than the disintegration of the catalyst or polymer particles during polymerization, i.e., the apparent density of the catalyst particles is lower than 2.32 g cm^−3^, which was assumed for the MgCl_2_ crystal. In the case of the non-modified catalysts (Cat50 and Cat200), the particle sizes were found to be 2–10 times greater than the theoretical sizes, indicating that further agglomeration proceeded during the polymerization. On the other hand, the deviation became unusually large for the modified catalyst system. Considering the dispersion stability of PA-Cat50 and PA-Cat200 in heptane, it was unlikely that the catalyst particles re-agglomerated during the polymerization. Rather, the plausible scenario was at the difficulty of polymer particles to be dispersed in ethanol against electrostatic force. In order to confirm this idea, a different mean of the dispersion was adopted. Figure 5B depicts a microscope image of dry powder (PA-PE50), which was physically dispersed and collected on a glass plate. The observed image evidenced the presence of very small particles that are well-separated from each other. Indeed, the particle size measurement based on the image analysis of vacuum-dispersed powder unveiled a much smaller particle size than that observed from light scattering (Figure 5C). PA-PE50 exhibited *D*_50_ of 1.7 μm with a narrow range of particle size distribution and particle solidity. From the particle shape analysis, PA-PE50 generally composed of two types of particles, the distorted sphere, and round shape. The former was found to be dominant with the range of the particle size close to the theoretical value (ca. 1 μm). It was believed that these distorted polymer particles were produced from primary catalyst particles, while some of them merged into a rounder shape with a bigger size during polymerization. In the case of R-PE, the polymer particles were also disintegratable due to a multigrain nature of the catalyst. This result is consistent with patent literature, where the popcorn-shape UHMWPE particles could be physically separated into fine particles by high speed shearing treatment (Suga et al., 1990). However, it could be noticed that the particle size distribution for R-PE was much broader than that of PA-PE50 and a major portion of particles was not disintegratable only by vacuum dispersion. These results evidenced that MgO/MgCl_2_/TiCl_4_ catalysts with nano-level dispersion allowed a direct access to microfine reactor powder.

Table 4 summarizes the DSC results. The melting temperature (*T*_m_) of as-obtained reactor powder (nascent form) was in the range of 140–143°C, while the *T*_m_ value was reduced to 132–135°C in the second heating (melt-crystallized form). The obtained values are consistent with literature reported for nascent and melt-crystallized UHMWPE having the molecular weight of 4.5 × 10^6^ g/mol (Rastogi et al., 2005, 2007). In fact, the *M*_v_ values of polymer powder obtained from PA-Cat50 and R-Cat were measured as 3.7 × 10^6^ and 4.4 × 10^6^ g mol^−1^, respectively. These results confirmed that the nano-dispersed MgO/MgCl_2_/TiCl_4_ catalysts enabled the production of PE having the molecular weight in the range of ultra-high molecular weight similar to a typical Ziegler-Natta catalyst. A higher *T*_m_ value for the nascent form with respect to that for the melt-crystallized form was explained by the difference in crystal topology, where the cooperative melting of several lamellae is required for the nascent form to adopt the random coil state (Rastogi et al., 2007). The crystallinity (*X*_c_) and the crystallization temperature (*T*_c_) for all of the samples were found to be in a similar range, and these values are typical for UHMWPE produced by Ziegler-Natta-type catalysts (Table 4; Joo et al., 2000; Schaller et al., 2015; Li et al., 2018; Tuskaev et al., 2018).

**Table 4:** DSC results.

**Sample**	**First heating** **(50**–**180**^**°**^**C)**			**Cooling** **(180–50**^**°**^**C)**		**Second heating** **(50–180**^**°**^**C)**		
	***T*_m_** **(°C)**	**Δ*H*_m_** **(J g^−1^)**	**$X_{\text{c}}^{\text{a}}$** **(%)**	***T*_c_** **(°C)**	**Δ*H*_c_** **(J g^−1^)**	***T*_m_** **(°C)**	**Δ*H*_m_** **(J g^−1^)**	**$X_{\text{c}}^{\text{a}}$** **(%)**
PA-PE50^b^	142.6	177.5	61.3	119.9	127.5	135.5	136.1	47.0
PA-PE200	140.2	173.6	60.0	120.4	141.4	135.0	139.1	48.1
PE50	140.2	185.0	63.9	122.6	125.4	132.5	150.7	52.1
PE200	140.2	192.3	66.5	121.8	140.9	133.7	149.4	51.7
R-PE^c^	142.8	176.5	61.0	119.2	137.7	135.8	143.1	49.4

a ΔH_100%_ = 289.3 J g^−1^ (ASTM F2625).

b M_v_ = 3.7 × 10^6^ g mol^−1^.

c *M_v_ = 4.4 × 10^6^ g mol^−1^*.

**Figure 6:**
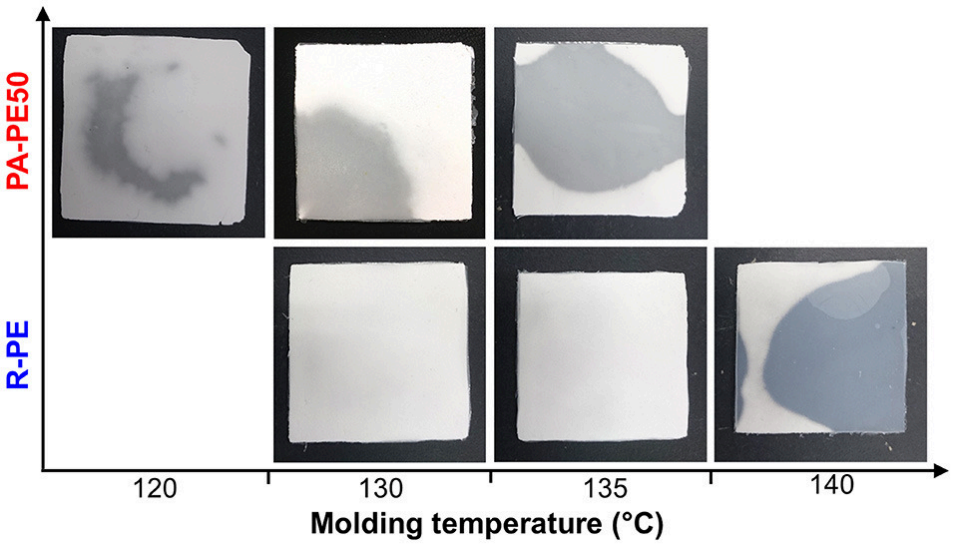
Compression-molded polymer reactor powder at different temperatures.

**Figure 7:**
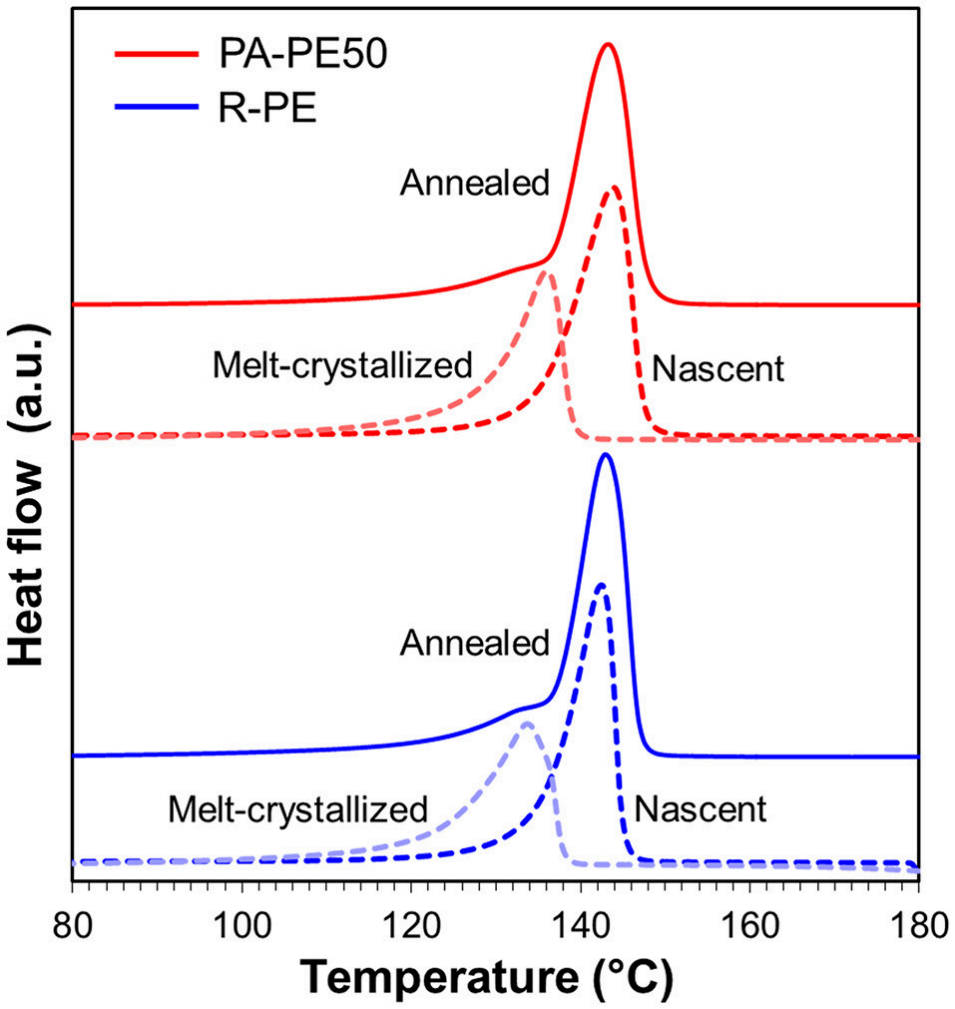
Melting behavior of polymer reactor powder after being annealed at 135°C for 60 min. Dashed lines are melting behavior for nascent and melt-crystallized forms as references.

In order to examine the processability of polymer reactor powder produced from the nano-dispersed catalyst, PA-PE50 was compressed into specimens at different molding temperatures. As shown in Figure 6, PA-PE50 started to fuse at 120°C as evidenced by the translucent region. On the other hand, R-PE required the molding temperature at least 140°C to start fusion. In general, the fusion of polymer powder involves physical processes such as melting, coalescence of particles, and crystallization (Hambir and Jog, 2000). In the case of the crystallization, the DSC results for the cooling (Table 4) revealed that PA-PE50 and R-PE samples have almost an identical crystallization temperature as well as a comparable crystallinity in the second heating. Hence, a significant difference in the crystallization behavior is unlikely. In regards to the polymer melting, though the applied molding temperatures were below *T*_m_ of polymer in the nascent form, a fraction of polymer might be already melted. Additional DSC measurements were conducted on the annealed samples to identify any differences for this fraction. The annealing temperature of 135°C was selected due to the following reasons: (i) Both of the PA-PE50 and R-PE samples melted around this temperature in the second heating, and (ii) this temperature represented the upper limit to obtain a clear disparity of the appearance between the two samples. Figure 7 shows the DSC curves of PA-PE50 and R-PE after being annealed at 135°C for 60 min. The melting peaks for the nascent and melt-crystallized forms are also given as references. In the case of the annealed samples, a shoulder appeared in addition to the melting peak for the nascent form. This shoulder was related to the detachment of chains from the surfaces (Rastogi et al., 2007), which indicated that a part of crystals already melted under the processing condition. However, judging from a comparable DSC profile for both of the samples, it was concluded that PA-PE50 and R-PE possessed a similar melting behavior at the applied molding temperature. Considering the similarity in the molecular weight, crystallinity, melting and crystallization behaviors, a lower fusion temperature for PA-PE50 as compared to R-PE was most plausibly originated from the coalescence among particles. Though the coalescence of PA-PE50 particles could not be visually observed by an optical microscope due to too small particles, it is believed that the fine structure of PA-PE50 provided a larger contact interface to promote the fusion across the interface during compression molding. Additionally, a smaller size of voids between adjacent primary polymer particles might accelerate the process of compaction by shortening the flow path for particle sliding or elastic flow to complete the void filling step in molding.

**Figure 8:**
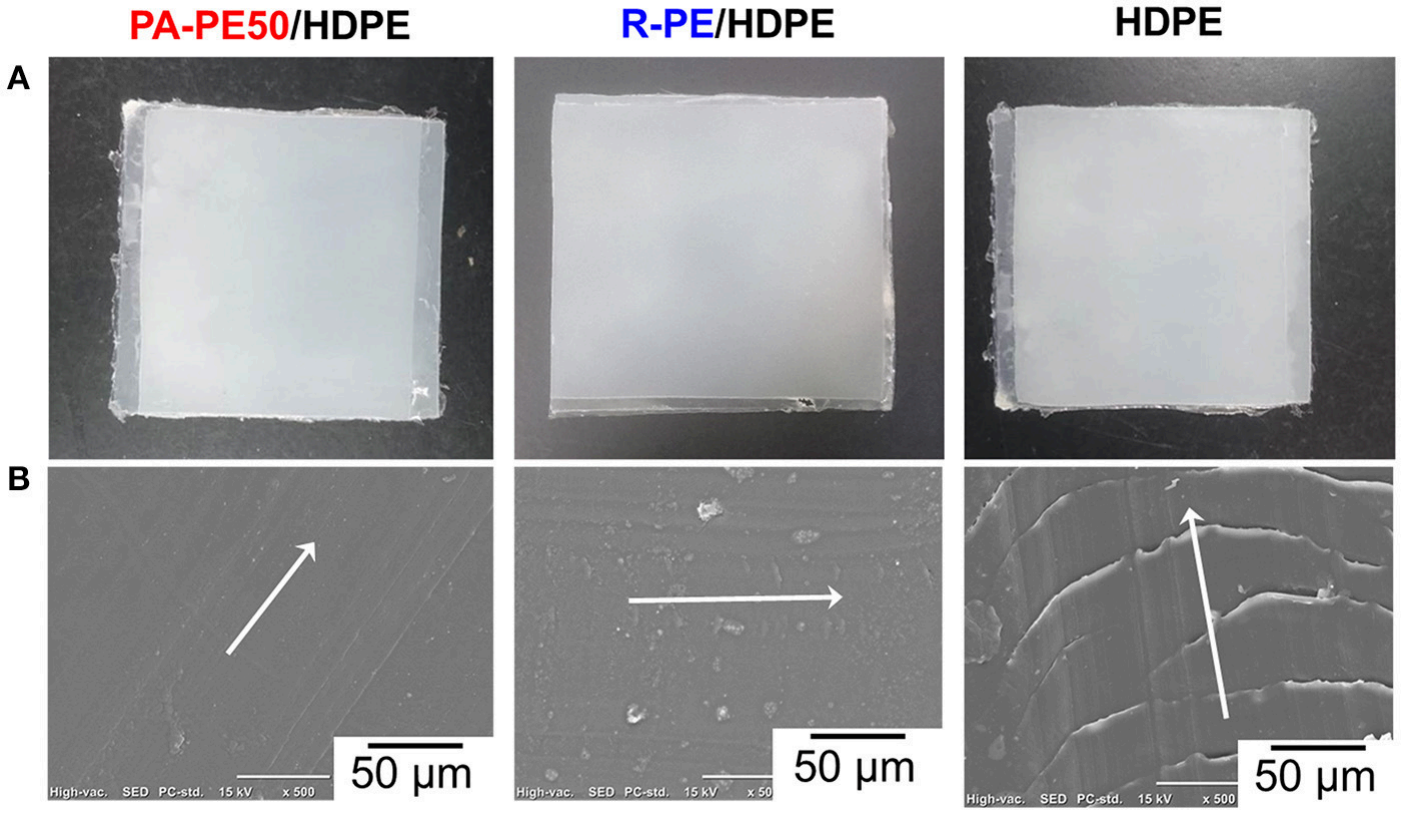
Scratch resistant of UHMWPE-coated HDPE: appearance of specimens **(A)**, and SEM images after the scratch test **(B)**. The arrows indicate the scratch direction.

As-obtained reactor powder was also used to form a scratch resistant coat on a HDPE plaque by compression molding. The appearance of the specimens and the microscopic view of the surface after introducing a scratch are illustrated in Figures 8A,B. It should be noted that the scratch was simply introduced using tweezers at an equivalent angle and force to preliminary observe the damage of the surface. As can be seen in Figure 8B, parabolic tracks were clearly visible for the original surface of HDPE. Contrary, coating the surface with both of PA-PE50 and R-PE powder noticeably introduced the scratch resistant property. In the case of PA-PE50 coating, no trace of the scratch was visible on the surface, while tiny parabolic tracks were observed for R-PE coating. The finished surface was also found to be much smoother for PA-PE50 than for R-PE. These results suggested that the fine structure of PA-PE50 allowed a better consolidation to improve the surface properties at a given processing temperature.

To the end, a potential of the nano-dispersed Ziegler-Natta catalyst is discussed in terms of the industrial process scale-up. The catalyst synthesis is comprised of two simple steps: A dispersion step (modification of MgO nanoparticles with an appropriate surfactant) and a catalyzation step (TiCl_4_ treatment of the modified MgO nanoparticles). MgO nanoparticles are not only producible by a variety of methods including the sol-gel, hydro/solvothermal, and even physical methods, but also widely commercially available. The choice of a proper surfactant is also done easily: Aprotic and neutral surfactants to accommodate with the TiCl_4_ treatment. Judging from the availability of the starting materials and the simplicity of the processes, facile scale-up is highly expected. The resultant catalyst can be used in a slurry polymerization process, similar to other Ziegler-Natta catalysts for the UHMWPE production, where the dispersibility of the nano-sized catalyst must be profitable in uniform feeding and polymerization. On the other hand, an electrostatic interaction for the fine UHMWPE powder and expected low bulk density may be a focus of a future research.

## Conclusions

A catalytic approach to produce fine polymer particles was proposed based on the exploitation of a nano-sized catalyst. In this work, a truly nano-dispersed Ziegler-Natta catalyst was firstly synthesized. The modification of MgO surfaces by a proper organic modifier improved the dispersion of MgO in a hydrocarbon solvent, so as to facilitate the formation of truly nano-dispersed MgO/MgCl_2_/TiCl_4_ core-shell catalysts. In ethylene polymerization, the MgO/MgCl_2_/TiCl_4_ catalysts afforded UHMWPE with the activity viable in an industrial point of view with a substantial reduction of the Cl content in the resultant polymer. Moreover, the polymer particle size measured based on a dry dispersion method was found to be in the range of 1-2 μm in agreement to the theoretical estimate. These extremely fine UHMWPE particles yielded several advantages in processing, such as a significantly lower fusion temperature and an improved consolidation in compression molding. In conclusion, the proposed approach facilitated several promising advantages in the production of UHMWPE, including simple preparation protocol, Cl-free, and direct access to microfine particles featured with better processability at a lower temperature.

## Author contributions

All authors listed have made a substantial, direct and intellectual contribution to the work, and approved it for publication.

### Conflict of interest statement

The authors declare that the research was conducted in the absence of any commercial or financial relationships that could be construed as a potential conflict of interest.
