# Supplementary material for: Nano-Dispersed Ziegler-Natta Catalysts for 1 μm-Sized Ultra-High Molecular Weight Polyethylene Particles
Source: Front Chem. 2018 Oct 30;6:524. doi: 10.3389/fchem.2018.00524 (PMC6232879; doi:10.3389/fchem.2018.00524)
Supplement: Supplementary file 1 [file Table_1.DOCX]

**Supplementary Information**

**Nano-dispersed Ziegler-Natta catalysts for 1 µm-sized ultra-high molecular weight polyethylene particles**

**Patchanee Chammingkwan, Yusuke Bando, Minoru Terano, Toshiaki Taniike***

Graduate School of Advanced Science and Technology, Japan Advanced Institute of Science and Technology, 1-1 Asahidai, Nomi, Ishikawa 923-1292, Japan

*** Correspondence:**Toshiaki Taniike
taniike@jaist.ac.jp

**
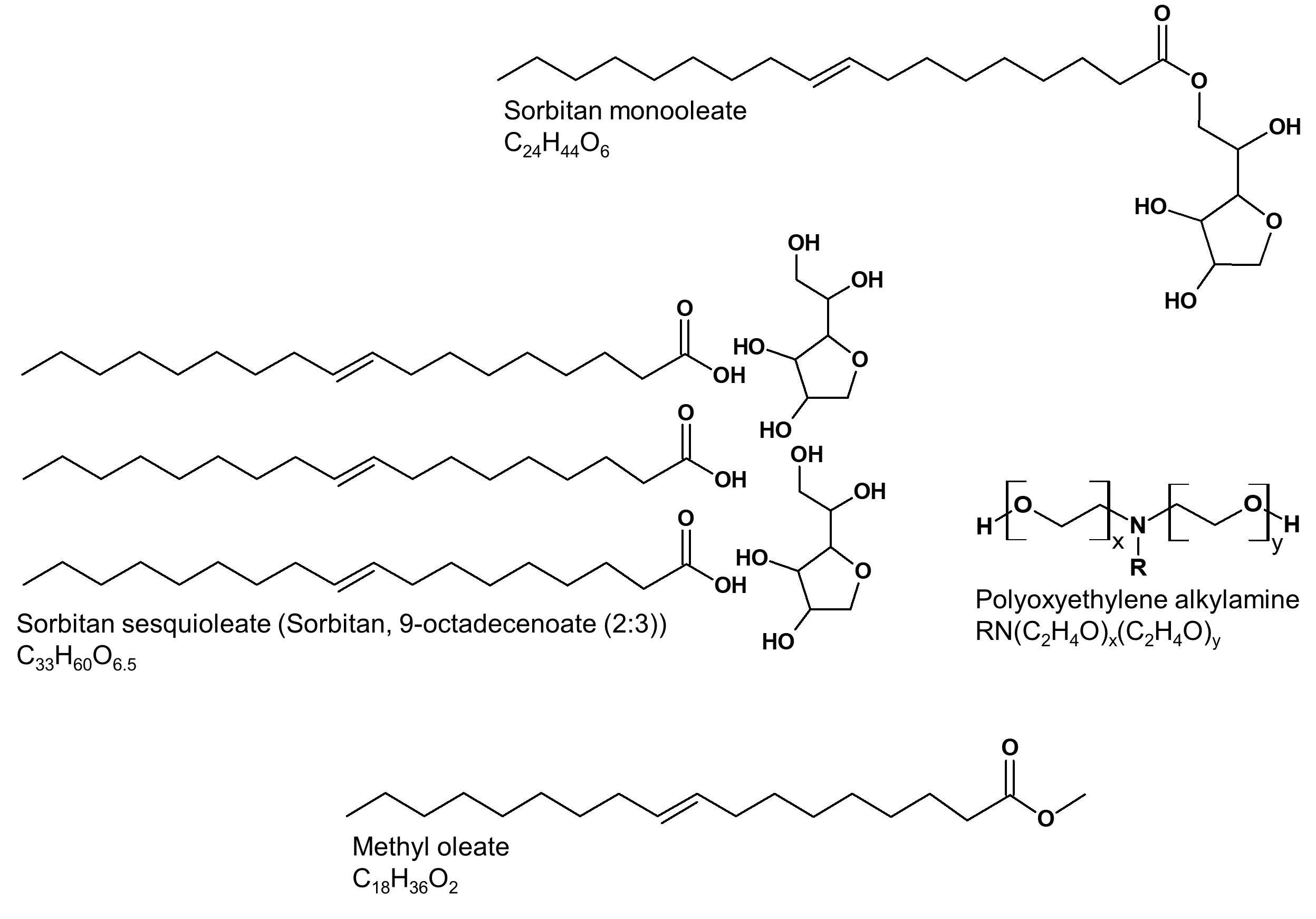
**

**Figure S1.** Chemical structures of organic modifiers.
